# Impact of psoriatic disease on women aged 18 to 45: Results from a multinational survey across 11 European countries

**DOI:** 10.1016/j.ijwd.2021.08.011

**Published:** 2021-08-28

**Authors:** Sandy R. McBride, Maria C. Fargnoli, Anne-Claire Fougerousse, Marta García Bustínduy, Louise Catton, Lerzan Senturk, Cécile Ecoffet, Jan Koren, Laura Andreoli, Laura C. Coates, Alice Titialii

**Affiliations:** a Department of Dermatology, Royal Free London NHS Foundation Trust, London, United Kingdom; b Department of Biotechnological and Applied Clinical Sciences, University of L'Aquila, L'Aquila, Italy; c Department of Dermatology, GEM ResoPso, Hôpital d'Instruction des Armées Bégin, Saint-Mandé, France; d Department of Internal Medicine, Dermatology and Psychiatry, Universidad de La Laguna, San Cristobal de La Laguna, Spain; e UCB Pharma, Slough, United Kingdom; f UCB Pharma, Brussels, Belgium; g EUROPSO, Polzela, Slovenia; h Department of Clinical and Experimental Sciences, University of Brescia, Brescia, Italy; i Unit of Rheumatology and Clinical Immunology, ASST Spedali Civili, Brescia, Italy; j Nuffield Department of Orthopaedics, Rheumatology and Musculoskeletal Sciences, University of Oxford, Oxford, United Kingdom; k International Federation of Psoriasis Associations, Stockholm, Sweden

**Keywords:** Psoriasis, psoriatic arthritis, women of childbearing age, family planning, mental health

## Abstract

**Background:**

Plaque psoriasis (PSO) is a long-term inflammatory condition that can cause concomitant joint symptoms (psoriatic arthritis [PsA]) in up to 30% of patients. The impact of psoriatic disease on disease outcomes and quality of life is greater in women than men.

**Objective:**

We evaluated the impact of psoriatic disease on women aged 18 to 45 years across Europe.

**Methods:**

Women aged 18 to 45 years with moderate to severe PSO, PsA, or PSO + PsA (PSO with progression to PsA) and prior biologic experience were recruited from market research panels and patient association groups of the International Federation of Psoriasis Associations, European Federation of Psoriasis Patient Associations, and Arthritis Ireland and asked to complete a survey. Questions covered social and psychological wellbeing, employment, and family planning. Question types included 5- or 7-point agreement scales, where the highest/lowest two ratings were considered agreement/disagreement, respectively. The results are reported as proportions of those who selected the answer, divided by overall respondents for each question. Women were not required to answer all questions.

**Results:**

Survey respondents (N = 573) had a diagnosis of PSO (n = 236), PsA (n = 173), or PSO + PsA (n = 164). Women self-reported similar mean scores for physical (57.0 of 100) and mental (59.0 of 100) health. A fifth (21%) had not achieved their desired career due to PSO/PsA; career dissatisfaction and increased sick leave were linked to poor mental health. Some women reported having a limited social life (33%), smaller families (34%), and being more likely to adopt children (27%) due to PSO/PsA. A quarter of women (27%) reported not understanding enough about PSO/PsA (nonmembers vs. members of patient association groups: 37% vs. 8%).

**Conclusion:**

Our findings highlight the considerable burden of psoriatic disease on women of childbearing age. Increased provision of information tailored to women, training for health care professionals, and shared decision-making between patients and health care professionals may help better support women with psoriatic disease.

**PUBLICATION IMPACT**

**What is known about this subject in regard to women and their families?**

• The impact of psoriatic disease on disease outcomes and quality of life are greater for women than men.
• The early onset of psoriatic disease results in diagnoses and treatments that typically overlap with a female patient‘s reproductive years and the start of their career.
• Women with psoriatic disease report greater psychological implications associated with their disease, compared with men.

**What is new from this article as messages for women and their families?**

• Despite previous or current biologic treatment, many surveyed women reported negative impacts on their social relationships, mental health and career.
• Family planning was a source of a variety of concerns, highlighting the need for patient education and support from healthcare providers.

## Introduction

Plaque psoriasis (PSO) is a long-term inflammatory condition that typically causes the appearance of raised, erythematous, and scaly lesions on the skin (National Psoriasis Foundation, 2020). Concomitant joint symptoms, including pain and swelling, occur in approximately 30% of patients, indicating the development of psoriatic arthritis (PsA; Mease et al., 2019). PSO can develop at any age, but most cases occur before the age of 35 years and women are often diagnosed slightly earlier than men (National Institute for Health and Care Excellence, 2012; Queiro et al., 2014; World Health Organization, 2016). Patients who develop concomitant PsA are usually diagnosed 5 to 10 years after the onset of PSO (Mease and Armstrong, 2014). The early onset of psoriatic disease (PSO and/or PsA) means that diagnosis and treatment are likely to overlap with a patient's reproductive years and may affect aspects of family planning, such as pregnancy and breastfeeding for women (De Simone et al., 2019). Disease onset is also more likely to coincide with the beginning of a patient's career and negatively affect earning potential and productivity (Ayala et al., 2014; De Simone et al., 2019; Gottlieb et al., 2019; Tillett et al., 2015).

Although it has been well-documented that psoriatic disease can negatively affect many aspects of patients’ lives, it has only recently been highlighted that women are disproportionately affected, compared with men (Hawro et al., 2017; Højgaard et al., 2018; LEO Innovation Lab, 2017; Murer et al., 2021). In addition to the reduced response to certain treatments and higher levels of symptoms, such as pruritus, the emotional and psychological impact of psoriatic disease also differs for women compared with men (Hawro et al., 2017; Højgaard et al., 2018; LEO Innovation Lab, 2017, 2018; Murer et al. 2021). Women with PSO report lower levels of happiness and higher levels of stress and loneliness than men, as well as higher levels of stigma, a predictor of reduced quality of life (QoL; Hawro et al. 2017; LEO Innovation Lab 2017).

Given the long-term nature of psoriatic disease and its greater impact on women, it is important to gain a better understanding of how the disease affects women's lives. Our findings highlight the challenges faced by women with psoriatic disease and illustrate the importance of a holistic approach to treatment, including shared decision-making and multidisciplinary support.

## Methods

### Study design

The survey was developed by Cello Heath and informed by a gap analysis of previous market research and published literature in this patient population. The draft survey was reviewed by UCB employees and representatives of the International Federation of Psoriasis Associations (IFPA) and European Federation of Psoriasis Patient Associations (EUROPSO) and piloted with four patients recruited via patient association groups. The questionnaire was adapted using patient feedback and distributed between June 2019 and January 2020.

The survey featured four themes: social life, psychological impact, career, and family planning. Patient demographics, including age group, disease severity, and family planning status, were collected. Questions included single or multiple response options; where 5- or 7-point agreement scales were given, the highest and lowest two values were considered agreement and disagreement, respectively. Other questions used statement-based Likert scales, where mental and physical health were rated on a scale from 1 to 100, with 100 indicating the best health possible. The full survey is available in Table S1.

### Patients

Patients were pre-screened for eligibility to ensure that they were female, aged 18 to 45 years, and had a diagnosis of PSO, PsA, or PSO + PsA. These eligibility criteria were chosen so that the results of this survey would focus on the key patient population, women of childbearing age. No control group participants were recruited. The diagnosis was required to be >1 year prior to survey completion, and although self-reported, the question specifically asked about diagnosis from a healthcare professional (HCP). Women were required to have prior or current experience with biologic treatment; this is usually prescribed for moderate to severe psoriatic disease, so this criterion was intended to ensure a certain level of disease severity. However, despite this criterion, women could self-report their disease severity as mild, moderate, or severe. If a patient did not meet ≥1 of the eligibility criteria, they were not invited to answer further questions.

Women from 11 European countries (Belgium, Finland, France, Germany, Greece, Ireland, Italy, the Netherlands, Spain, Sweden, and the United Kingdom) were recruited through a third-party market research panel (Dynata) and patient association groups (from IFPA, EUROPSO, and Arthritis Ireland). Patients were contacted through telephone conversations, newsletters, and social media. The target number of respondents was 600, to comprise approximately 250 women sourced from patient association groups and 350 to 400 women from market research panels. Women with PSO and PsA were recruited at a target ratio of 1:1. All collected data were anonymized so that patient identification was not possible.

### Statistical analyses

Data are reported as percentages of women (number of respondents selecting each answer divided by the total number of respondents to that question). Women were not required to answer every question, so missing respondents were not included in the proportions. For questions rating mental and physical health, mean scores are presented. Z tests comparing each disease group with another were used to indicate significance at the 95% level. The *p*-values are nominal and should be interpreted with caution.

## Results

### Patient disposition and characteristics

Approximately a third of the contacted women responded, although an initial pre-screen for diagnosis was conducted before their invitation. Of these respondents, 573 women were eligible and provided survey responses.

Overall, 41% of women (n = 236) with prior or current biologic treatment self-classified as having PSO, 30% (n = 173) as PsA, and 29% (n = 164) as both PSO and PsA (PSO + PsA; Table 1). Women in the PSO + PsA group were generally older than those in the separate PSO and PsA groups, and more than half of all women reported that their doctor classified their symptoms as moderate (Table 1).

**Table 1:** Baseline patient characteristics and demographics

n (%), unless otherwise stated	PSO (n = 236)	PsA (n = 173)	PSO + PsA (n = 164)
**Age group, y**			
18–25	40 (17)	41 (24)	14 (9)
26–35	103 (44)	65 (38)	61 (37)
36–45	93 (39)	67 (39)	89 (54)
**Time since diagnosis, y**			
1–3	70 (30)	68 (39)	34 (21)
>3–5	63 (27)	54 (31)	41 (25)
>5–10	32 (14)	33 (19)	28 (17)
>10	71 (30)	18 (10)	61 (37)
**Disease severity**^a^			
Mild	20 (8)	15 (9)	13 (8)
Moderate	138 (58)	118 (68)	85 (52)
Severe	74 (31)	35 (20)	57 (35)
Do not know/unsure	4 (2)	5 (3)	9 (5)
**Pre-existing conditions**^b^**^,^**^c^			
Depression/anxiety/other mental health illness	51 (22)	62 (36)	52 (33)
Migraine/headache	44 (19)	34 (20)	38 (24)
High blood pressure/hypertension	29 (13)	24 (14)	23 (14)
Obesity	31 (13)	18 (11)	22 (14)
Diabetes	30 (13)	14 (8)	14 (9)
Asthma	20 (9)	17 (10)	23 (14)
**Treatments currently taken**^d^			
1	64 (27)	42 (24)	49 (30)
2	72 (31)	46 (27)	46 (28)
3	46 (19)	47 (27)	36 (22)
4	28 (12)	18 (10)	16 (10)
5+	26 (11)	20 (12)	17 (10)
**Family planning status**^c^			
Trying to conceive/expect to start soon	35 (16)	23 (14)	21 (13)
Currently pregnant	12 (5)	9 (5)	5 (3)
Given birth in the last 5 y	71 (32)	54 (32)	48 (30)
None of the above	107 (48)	83 (49)	85 (53)
**Country of residence**			
United Kingdom	30 (13)	29 (17)	11 (7)
Germany	24 (10)	26 (15)	16 (10)
France	41 (17)	16 (9)	13 (8)
Italy	38 (16)	14 (8)	19 (12)
Spain	40 (17)	1 (1)	36 (22)
The Netherlands	16 (7)	19 (11)	4 (2)
Greece	9 (4)	10 (6)	9 (5)
Belgium	14 (6)	8 (5)	7 (4)
Finland	13 (6)	9 (5)	13 (8)
Sweden	5 (2)	21 (12)	17 (10)
Ireland	6 (3)	20 (12)	19 (12)

PsA, psoriatic arthritis; PSO, psoriasis; y, years.

a As described by the women's doctor, self-reported by women at the time of survey.

b Those reported were present in ≥10% of the total group.

c Not all women responded to these questions (pre-existing conditions: PSO = 230, PsA = 171, PSO + PsA = 159; family planning status: PSO = 225, PsA=169, PSO + PsA =159).

d Relates to any treatments taken at the time of the survey specifically for the relevant condition, including phototherapy, topical treatments, and various biologics.Here, a diagnosis of PSO + PsA refers to PSO with progression to PsA.

### Impact on daily life and social interactions

**Fig. 1:**
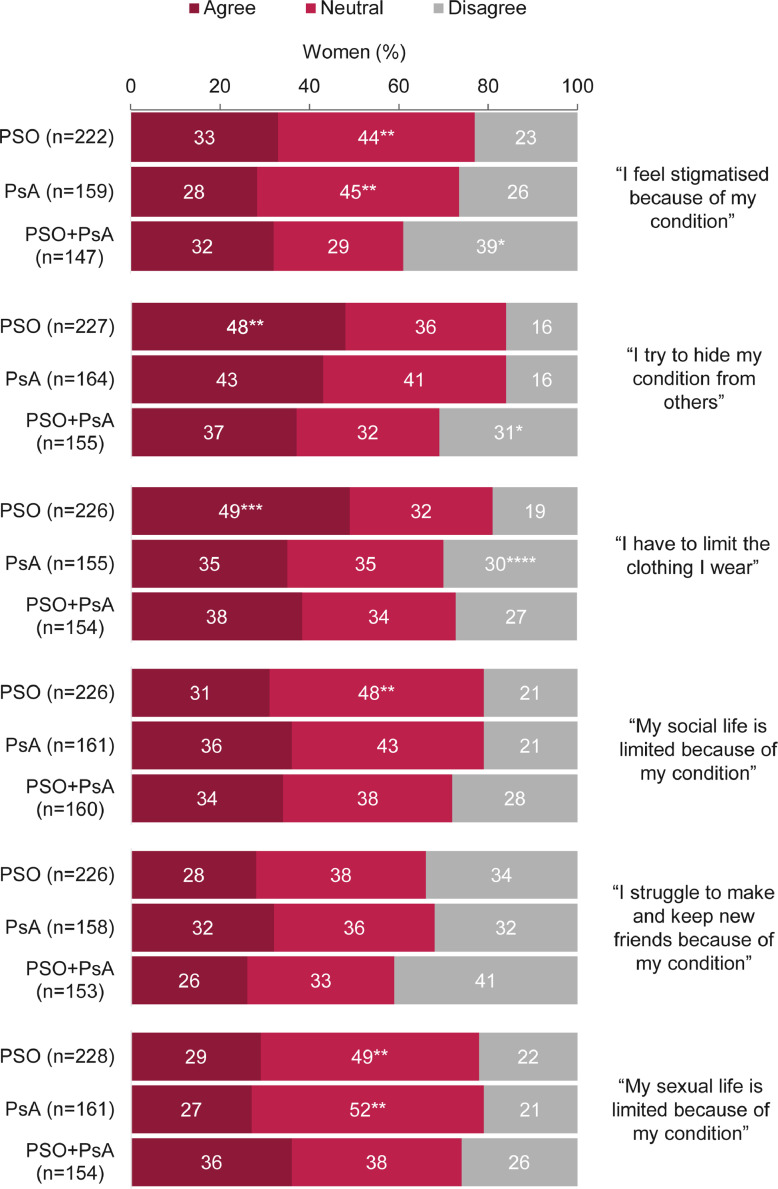
Agreement with statements on the social effects of diagnosis. *Significant when compared with the psoriasis (PSO) and psoriatic arthritis (PsA) disease groups (*p* < .05). ^⁎⁎^Significant when compared with the PSO + PsA disease group (*p* < .05). ^⁎⁎⁎^Significant when compared with the PsA and PSO + PsA disease groups (*p* < .05). ^⁎⁎⁎⁎^Significant when compared with the PSO disease group (*p* < .05). Agreement was defined as a rating of 6 or 7 on a 7-point Likert scale when asked: “How well do each of the following statements apply to you?”; “neutral” indicates a rating of 3 to 5 and “disagree” indicates a rating of 1 or 2. Percentages may not add up to 100 due to rounding. Data are reported as percentages of women (the number of respondents selecting each answer divided by the total number of respondents to that question). Women were not required to answer every question, so missing respondents were not included in the proportions. Overall, 573 women were asked this question.

Of all women (N = 573), 31% (165 of 528) agreed that they felt stigmatized, and 42% (223 of 535) reported limiting their clothing due to psoriatic disease; this was significantly more common in women with PSO compared with those with PsA or PSO + PsA (*p* < .05; Fig. 1). Although the majority of women believed their treatment allowed them to live a somewhat normal or completely normal life, almost half (45%; 252 of 565) agreed that they were worried about the long-term impact of treatment; this was significantly higher in women with PSO + PsA (*p* < .05; Fig. S1).

A third of women (33%; 183 of 547) reported limitations to their social life because of their condition (Fig. 1). Overall, 29% of women (154 of 537) agreed that they struggled with making and maintaining friendships, and 30% (165 of 543) agreed that their sexual life was limited because of their diagnosis (Fig. 1). Women with recent diagnoses (n = 330; ≤5 years since diagnosis) were more likely than those with longer-term diagnoses (n = 243; >5 years since diagnosis) to report a negative impact on platonic and intimate relationships (Fig. S2).

### Psychological impact

The overall mean rating for physical health across all women was 57.0 of 100 (n = 558). A similar mean score of 59.0 of 100 was reported for mental health (n = 562), although these questions had different respondents. The mean scores for physical and mental health were similar across diagnoses (Fig. S3).

Approximately a third of women (34%; 194 of 566) reported feeling pessimistic about the future; this was more common among women with PSO + PsA (41%) compared with the PSO or PsA groups (PSO: 30%; PsA: 34%; Fig. S4A). When asked about a normal day living with their condition, 45% of women (254 of 565) reported positive emotions (including feeling courageous and resilient; Fig. S4B), and 78% (440 of 565) reported negative emotions; this was comparable across disease groups. Negative emotions were reported by more women during disease flares (skin and/or joint related; 97%; 546 of 565).

### Career

Employment status was similar across diagnoses, with 55% of women (312 of 568) in full-time employment and others in part-time employment, at home by choice, on long-term sick leave, unemployed, or other (students and self-employed; Fig. S5).

**Fig. 2:**
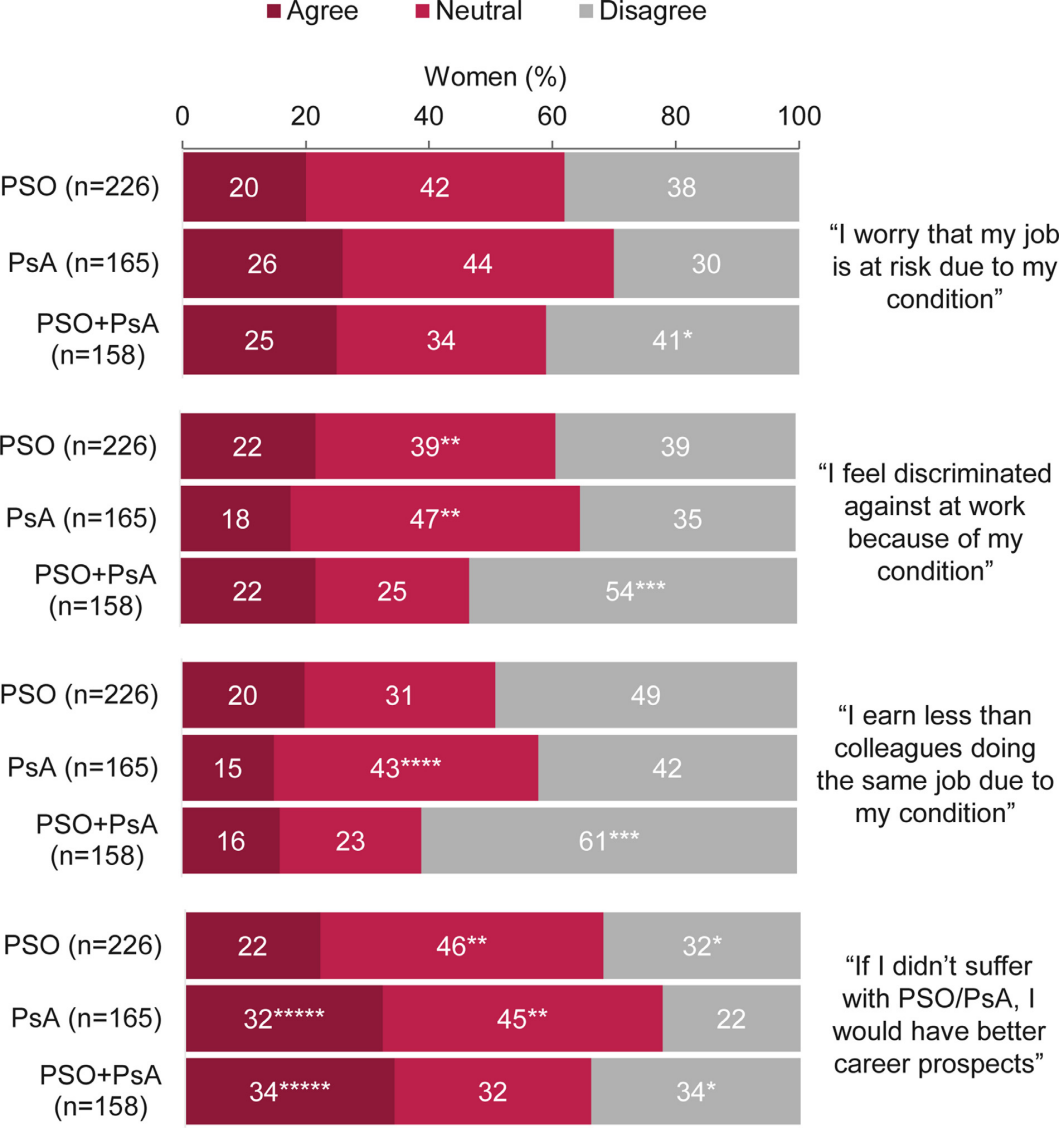
Agreement with statements relating to employment. *Significant when compared with the psoriatic arthritis (PsA) disease group (*p* < .05). ^⁎⁎^Significant when compared with the psoriasis (PSO) + PsA disease group (*p* < .05). ^⁎⁎⁎^Significant when compared with the PSO and PsA disease groups (*p* < .05). ^⁎⁎⁎⁎^Significant when compared with the PSO and PSO + PsA disease groups (*p* < .05). ^⁎⁎⁎⁎⁎^Significant when compared with the PSO disease group (*p* < .05). Percentages may not add up to 100 due to rounding. Data are reported as percentages of women (the number of respondents selecting each answer divided by the total number of respondents to that question). Women were not required to answer every question, so missing respondents were not included in the proportions. Overall, 573 women were asked this question.

Women who were employed full-time, part-time, or self-employed (n = 453) reported 26.5 days per year sick leave, on average; this was highest for those with PSO + PsA (PSO: 20.6 [n = 191]; PsA: 29.5 [n = 128]; PSO + PsA: 32.2 [n = 134]). Due to their diagnosis, approximately a quarter of women (23%; 129 of 549) felt worried that their job was at risk, 21% (114 of 549) felt discriminated against at work, and 18% (97 of 549) perceived that they earned less than colleagues doing the same job (Fig. 2). Women with PsA and PSO + PsA were significantly more likely than those with PSO to report that they would have better career prospects if they did not have psoriatic disease (*p* < .05; Fig. 2). A fifth of women (21%; 115 of 551) reported not having their desired career and that their condition was a major limiting factor in this, which was reported by significantly more women with PSO + PsA and PsA than women with PSO (PSO: 12% [27 of 223]; PsA: 24% [40 of 169]; PSO + PsA: 30% [48 of 159]; *p* < .05).

Career dissatisfaction was linked to poor mental health, with those who were unhappy with their career rating their mental health as 52 of 100 on average, compared with 64 of 100 for those who were happy with their career. Similarly, those who reported a higher number of sick days had a lower mean mental health score (>30 days [n = 76]: 54 of 100; 1–5 days [n = 78]: 62 of 100).

### Family planning

Half of all women surveyed (50%; 278 of 553) reported experience with family planning, with 14% (79 of 553) trying to conceive or expecting to start soon, 5% (26 of 553) currently pregnant, and 31% (173 of 553) who had given birth in the last 5 years (Table 1).

**Fig. 3:**
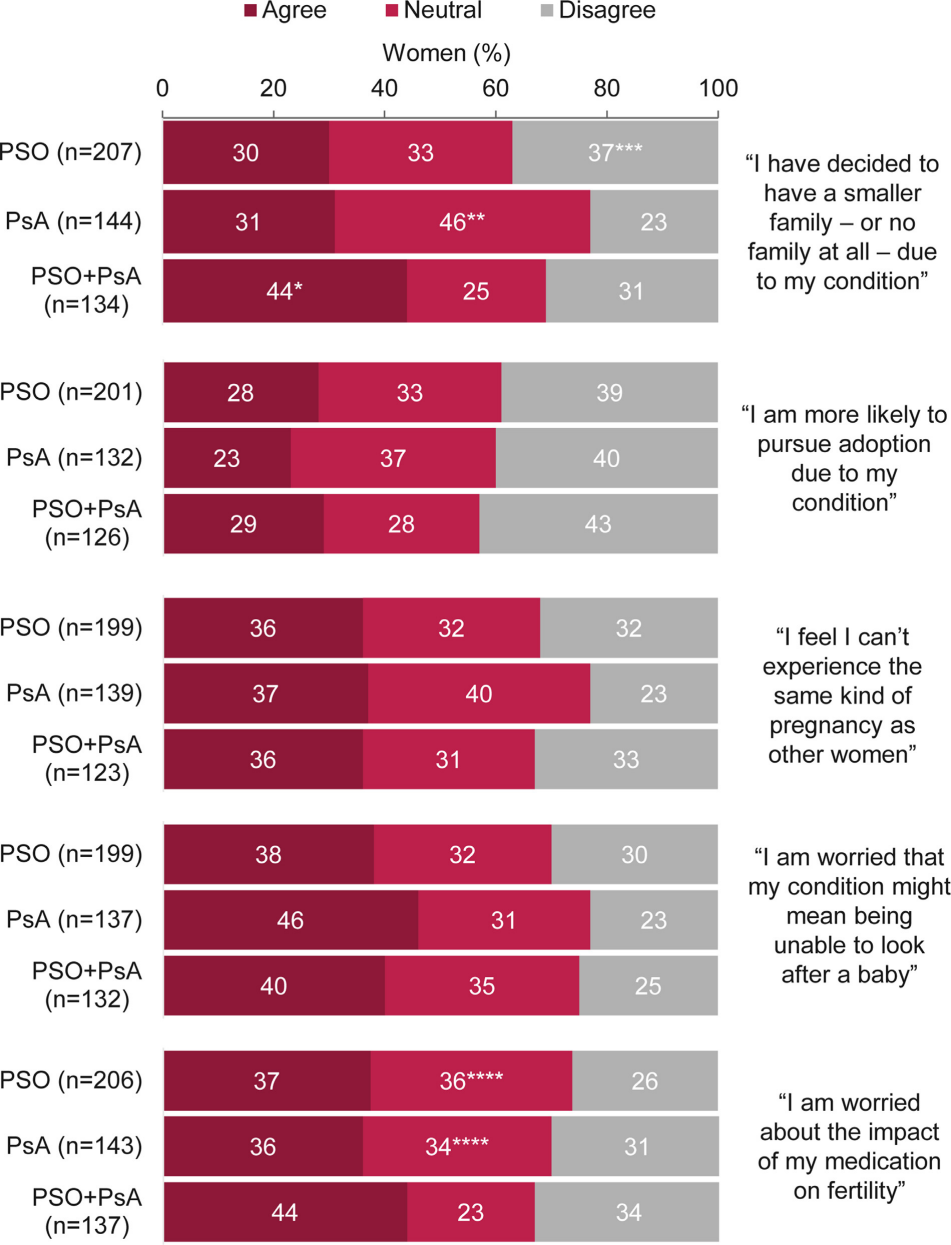
Agreement with statements relating to family planning. *Significant when compared with the psoriasis (PSO) and psoriatic arthritis (PsA) disease groups (*p* < .05). ^⁎⁎^Significant when compared with the PSO and PSO + PsA disease groups (*p* < .05). ^⁎⁎⁎^Significant when compared with the PsA disease group (*p* < .05). ^⁎⁎⁎⁎^Significant when compared with the PSO + PsA disease group (*p* < .05). Agreement was defined by a rating of 6 or 7 on a 7-point Likert scale when asked: “How well do each of the following statements describe you?”; “neutral” indicates a rating of 3 to 5 and “disagree” indicates a rating of 1 or 2. Percentages may not add up to 100 due to rounding. Data are reported as percentages of women (the number of respondents selecting each answer divided by the total number of respondents to that question). Women were not required to answer every question, so missing respondents were not included in the proportions. Overall, 573 women were asked this question.

More than a third of all women (34%; 167 of 485) had decided to have a smaller family (or no children) because of their condition; this was significantly higher among women with PSO + PsA compared with those with PSO and PsA (*p* < .05; Fig. 3). Overall, 11% (59 of 525) were childless and had decided to remain so (excluding those who selected “prefer not to answer”), and more than a quarter (27%; 124 of 459) were more likely to pursue adoption because of their condition. Commonly reported concerns included women's ability to experience the same kind of pregnancy as other women and look after a baby, as well as the effect of medication on fertility (Fig. 3).

**Fig. 4:**
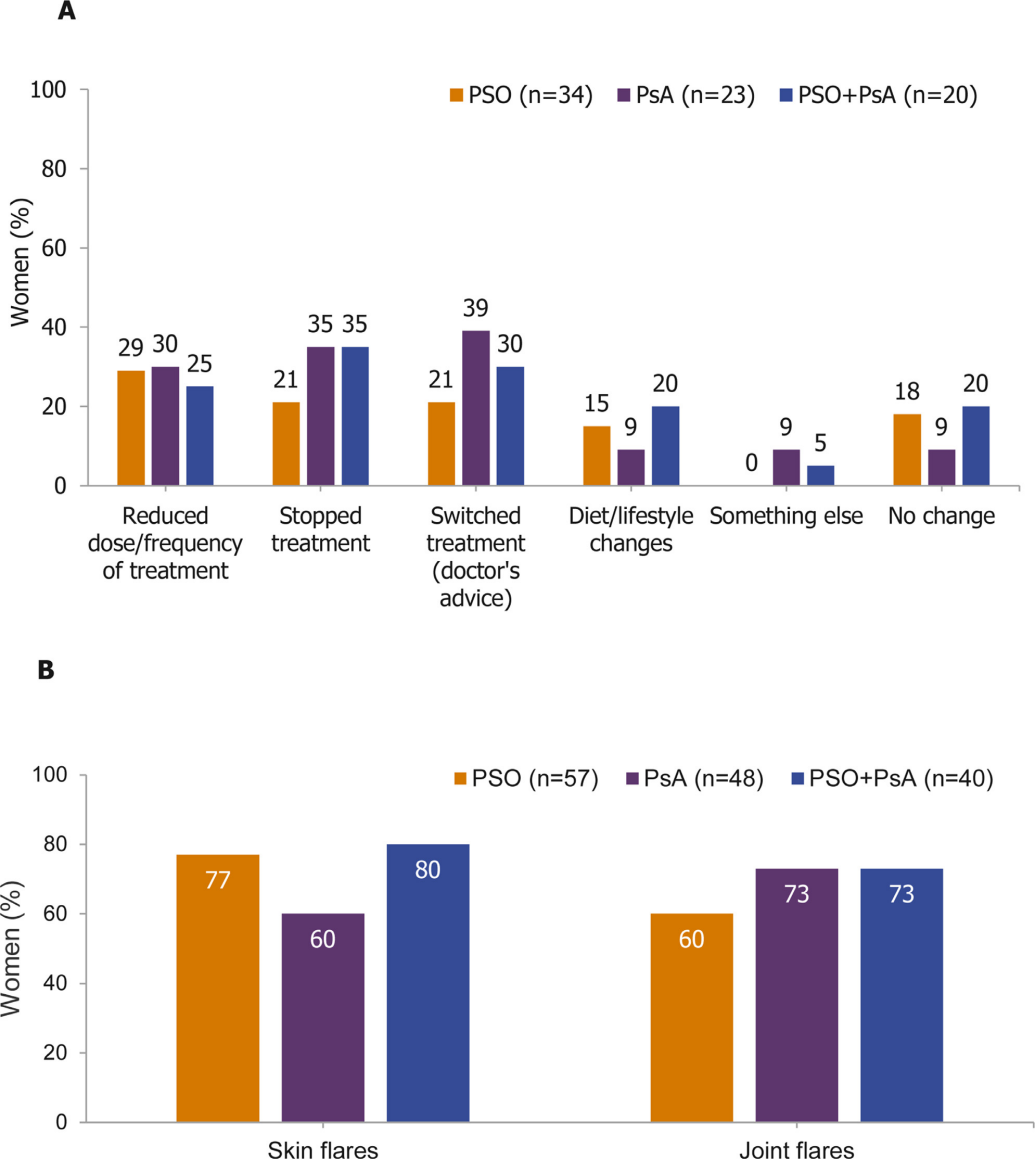
(A) Changes to treatment during pregnancy reported by women and (B) the percentage of women reporting skin and joint flares during pregnancy. Data are reported as percentages of women (the number of respondents selecting each answer divided by the total number of respondents to that question). Women were not required to answer every question, so missing respondents were not included in the proportions. Overall, 79 women were asked the question featured in part A and 199 women were asked that in part B of this figure. For both questions, women could select multiple answers.

Of those trying to conceive at the time of completing the survey, more than half (57%; 44 of 77) had reduced the dose or frequency of their treatment or switched to another treatment, and almost a third (29%; 22 of 77) had stopped treatment altogether (Fig. 4A). Among women who were pregnant or had given birth in the last 5 years, 72% (105 of 145) reported having experienced skin flares and 68% (98 of 145) joint flares during their pregnancy (Fig. 4B). Similarly, 68% (109 of 161) of those who had given birth in the last 5 years had experienced disease flares since giving birth.

### Patient support

More than half of all women (57%; 321 of 567) reported a moderate or low level of support from HCPs, and 55% (105 of 192) of those who were pregnant or had given birth during the last 5 years reported a moderate or low level of support from the HCPs managing their condition during pregnancy (Fig. S6A). When all women were asked about support services, many reported wanting support relating to stress relief, mental health, and fitness (Fig. S6B).

**Fig. 5:**
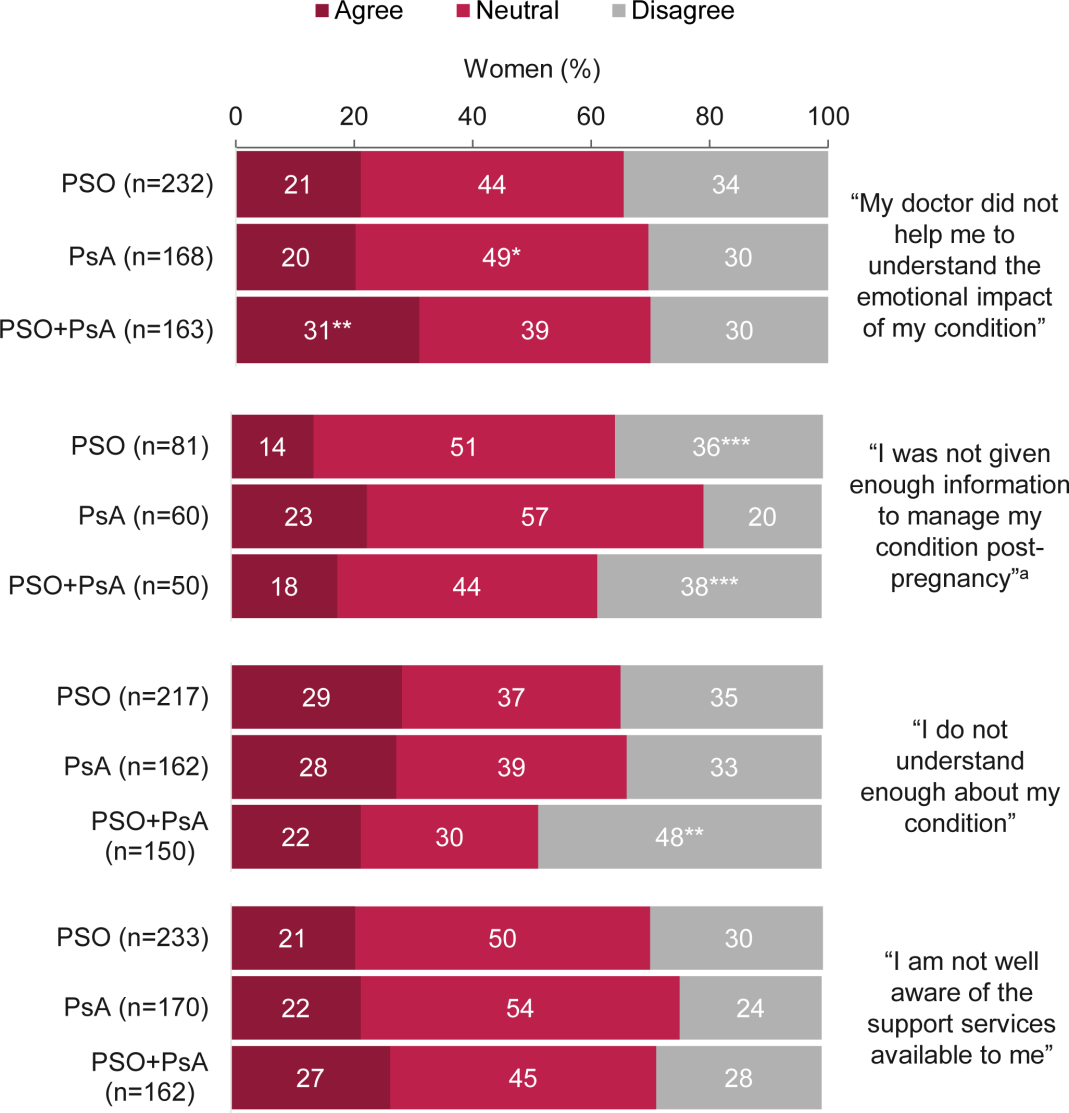
Agreement with statements referring to support and disease awareness. *Significant when compared with the psoriasis (PSO) + psoriatic arthritis (PsA) disease group (*p* < .05). ^⁎⁎^Significant when compared with the PSO and PsA disease groups (*p* < .05). ^⁎⁎⁎^Significant when compared with the PsA disease group (*p* < .05). ^a^Women answering this question were currently pregnant or had given birth in the last 5 years (n = 199). Data are reported as percentages of women (the number of respondents selecting each answer divided by the total number of respondents to that question). Women were not required to answer every question, so missing respondents were not included in proportions. Overall, 573 women were asked these questions. Agreement was defined by a rating of 6 or 7 on a 7-point Likert scale when asked: “How well do each of the following statements apply to you?”; “neutral” indicates a rating of 3 to 5 and “disagree” indicates a rating of 1 or 2. Percentages may not add up to 100 due to rounding.

More than two thirds (69%; 131 of 191) of those who were pregnant or had given birth during the last 5 years did not feel that they were given enough information regarding postpregnancy disease management (Fig. 5). When all women were asked about their disease-specific knowledge, 27% (141 of 529) reported that they did not understand enough (Fig. 5). This was considerably higher for those who were not members of patient association groups (35%; 126 of 355) compared with members (7%; 15 of 208). Feeling in control was linked to disease-specific knowledge; 60% of women who reported that they did not understand enough about psoriatic disease did not feel in control of their condition (vs. 16% who felt in control but did not understand enough).

## Discussion

This study indicates that women with psoriatic disease may continue to experience many challenges due to their disease, despite receiving, or having previously received, biologic treatment. Our results highlight that many diverse aspects of a woman's life may be affected, including social and psychological wellbeing, career, and family planning.

A high proportion of surveyed women reported feeling stigmatized due to psoriatic disease, and many reported an impact on mental health. More than three quarters of women reported negative emotions, including fatigue, anxiety, and frustration, on a normal day, increasing to almost all women during a disease flare. This aligns with the results of previous studies reporting that PSO leads to feelings of stigmatization, predominantly reported by women, and that PsA can lead to increased rates of anxiety and depression and reduced social functioning, all of which negatively affect mental wellbeing (McDonough et al., 2014; Hawro et al., 2017; Salaffi et al., 2009).

Patient responses suggested that recently diagnosed psoriatic disease has a greater impact on social relationships than longer-term diagnoses, although recently diagnosed patients were younger than those with longer-term diagnoses (64% vs. 47% for ≤35 years, respectively). This may support previous reports that loneliness and low self-esteem decrease with age in women with psoriatic disease, or indicate differences in social circumstances (McBride, 2019). Nevertheless, this highlights the importance of emotional support, particularly for women diagnosed at a younger age, because they are more likely to be vulnerable to negative emotions and social difficulties.

Our observations also indicate the potential impact of psoriatic disease on women's careers, with the average annual number of sick leave days for surveyed women far exceeding an estimate for employees in the European Union (26.5 vs. 11.9 days, including other health conditions; World Health Organization, 2016). However, sick days in this study were not documented as being related to PSO or PsA and may have been influenced by general illness or other comorbidities. Previous reports suggest that psoriatic disease can also reduce productivity, which may be further affected by depression and anxiety associated with the condition (LEO Innovation Lab, 2018; Tillett et al., 2015). These factors are likely to influence the feelings of career dissatisfaction and discrimination that many women reported in this study.

Family planning and childbearing aspirations were observed to be affected by psoriatic disease in this survey, with many women agreeing that their diagnosis led to them having a smaller family. This decision may be influenced by worries about caring for a baby or the effect of medication on fertility during pregnancy. With psoriatic disease influencing important family planning decisions for many women, including changes to treatment, HCPs are an important resource for information and support. A high proportion of women also reported disease flares during and after pregnancy, indicating potential worsening of their condition at this time. Many women reported a low or moderate level of support from HCPs while pregnant or attempting to conceive, supporting studies in other long-term inflammatory conditions that suggested an unmet need for patient information and a low awareness of relevant treatment guidelines among HCPs treating pregnant patients (Ackerman et al., 2015; Andreoli et al., 2019; Maccari et al., 2019; 2020; Nelson-Piercy et al., 2019). Commonly used guidelines, such as the 2012 European Alliance of Associations for Rheumatology guidelines for PsA treatment, often do not contain specific advice on treatment during pregnancy, and although the European Alliance of Associations for Rheumatology has separately published points to consider for the use of antirheumatic drugs before and during pregnancy, comparable guidelines for the treatment of pregnant women with dermatological diseases are lacking (Gossec et al., 2012; Götestam Skorpen et al., 2016; Gottlieb et al., 2019).

Improvements in shared decision-making between patients and HCPs could facilitate more integrated treatment pathways for women with psoriatic disease, potentially addressing the high proportion of women in this survey who reported a low or moderate level of general support from HCPs. These results may indicate a lack of open communication between patients and HCPs, supporting the results of previous studies in psoriatic disease (Gottlieb et al., 2019; Maccari et al., 2019; 2020). Improved training for HCPs focusing on facilitating discussions with patients about their treatment preferences and concerns could help alleviate some negative outcomes reported in this survey. Moreover, our findings highlight the importance of patient education to allow patients a greater feeling of control, which can be supported by HCPs and patient association groups.

The strengths of this study are that the survey provided a thorough exploration of the impact of psoriatic disease on the lives of women of childbearing age; this was achieved by the inclusion of several themes, each comprising many questions and several follow-up questions. Women were recruited from 11 different countries across Europe, resulting in data that are largely generalizable to many populations across the continent.

However, the study has some limitations preventing strong causal conclusions. Self-reporting may introduce bias, particularly because medical records were not reviewed and women were asked to confirm details provided by their doctor, relying on accurate recall and reporting. Although the biologic treatment inclusion criteria confirmed a threshold of disease severity, this may indicate that the survey group had more severe symptoms than the general patient population. Survey response may also indicate a high-impact patient, so reporting bias should be considered. The scope of this study did not extend to the effect of different localhealthcare systems on patient experience; this may be evaluated in future publications. As with other survey-based studies, ambiguity and misinterpretation of questions must be considered. The sample size was limited in some sections, particularly family planning and employment. The survey was aimed at our defined high-unmet-need patient population; due to this, there are no directly comparable results from control groups, limiting definitive comparisons. These data were collected prior to the COVID-19 pandemic, so responses should be considered as reflective of patient opinion at the time of survey completion.

## Conclusion

Our findings highlight the burden of psoriatic disease for many women of childbearing age, despite biologic treatment. Patient education was observed to play a significant role in women feeling in control of their condition, which can be assisted by patient association groups. Improved training for HCPs treating psoriatic disease is recommended to facilitate open discussions with patients and promote shared decision-making, particularly given that many women choose to alter or stop treatment during pregnancy. Given the relatively young age of many women with psoriatic disease and the evident impact on their daily lives, early diagnosis, treatment, and the provision of accessible support are crucial.

## Declaration of Competing Interest

Sandy R. McBride has received consultancy and/or speaker's fees from AbbVie, Almirall, Amgen, Celgene, Eli Lilly, Janssen, Novartis, and UCB Pharma and grant/research support from AbbVie and Celgene. Maria C. Fargnoli has served on advisory boards and received honoraria for lectures and research grants from Abbvie, Almirall, Celgene, Eli Lilly, Galderma, Janssen, Leo Pharma, Medac Pharma, MSD, Mylan, Novartis, Pierre Fabre, Pfizer, UCB Pharma, Roche, Sanofi-Genzyme, and Sun Pharma. Anne-Claire Fougerousse has received consultancy and/or speaker's fees from AbbVie, Janssen, Lilly, Novartis, and UCB Pharma. Marta García Bustínduy has been invited to give talks, contribute to studies and investigations, and received support to attend meetings and symposia from AbbVie, Almirall, Celgene, Janssen Cilag, Leo Pharma, Lilly, Novartis, and UCB Pharma. Louise Catton, Lerzan Senturk, and Cécile Ecoffet are employees of UCB Pharma and own shares. Laura Andreoli has received consultancy and/or speakers’ fees from Eli Lilly, Glaxo Smith Kline, Novartis, and UCB Pharma. Laura C. Coates has received consultancy fees from AbbVie, Amgen, Biogen, Boehringer Ingelheim, Celgene, Eli Lilly, Gilead, Janssen, Medac, Novartis, Pfizer, and UCB Pharma and research grants from AbbVie, Amgen, Celgene, Janssen, Novartis, and Pfizer. All other authors have no conflicts of interest to declare..

## Appendix Supplementary materials

Image, application 1
