# Supplementary material for: Impact of psoriatic disease on women aged 18 to 45: Results from a multinational survey across 11 European countries
Source: Int J Womens Dermatol. 2021 Aug 28;7(5Part B):697–707. doi: 10.1016/j.ijwd.2021.08.011 (PMC8714576; doi:10.1016/j.ijwd.2021.08.011)
Supplement: Supplementary file 1 [file mmc1.pdf]

Figure S1. Agreement with statements relating to treatment

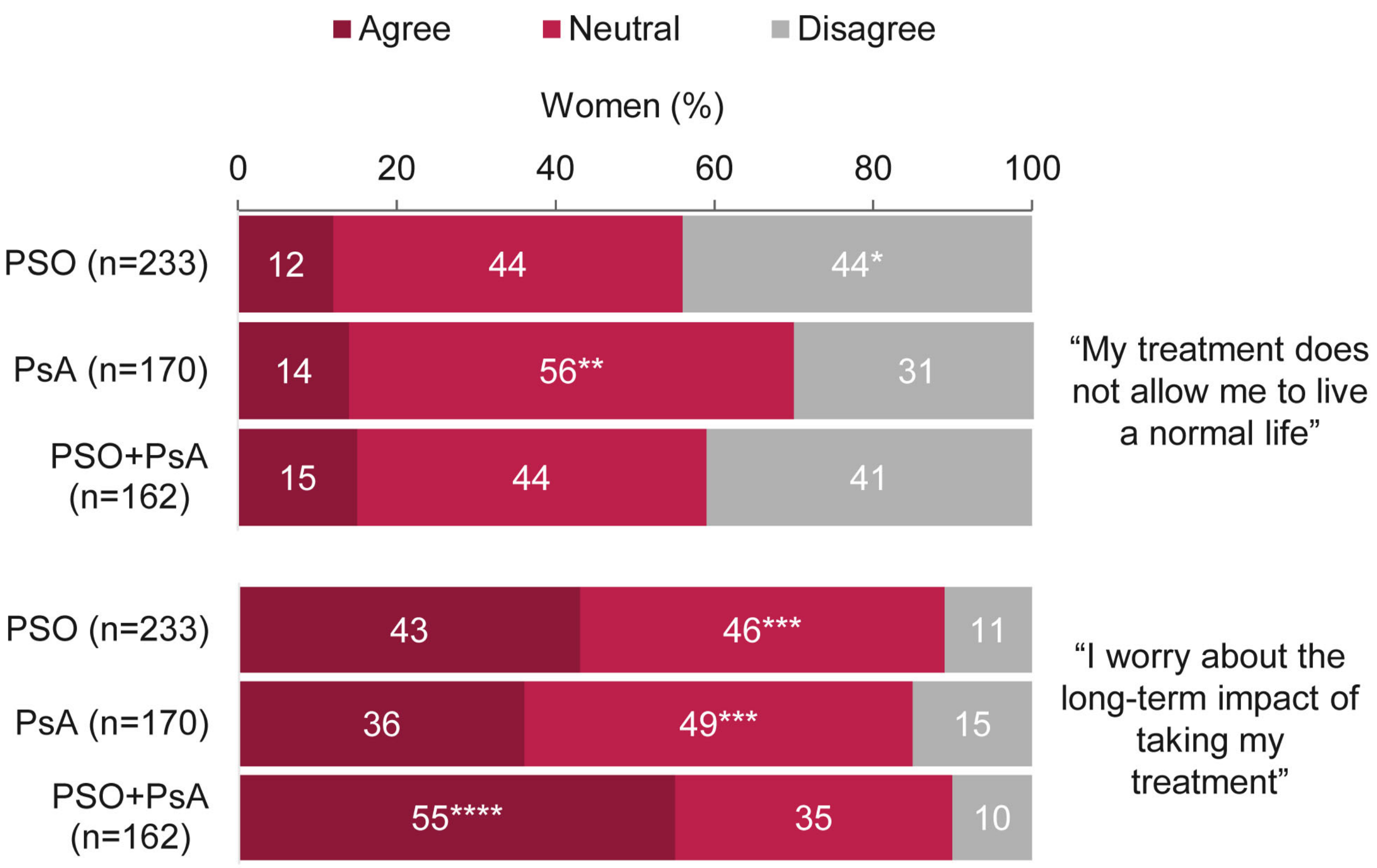

*Significant when compared to the PsA group; **Significant when compared to the PSO and PSO+PsA groups; ***Significant when compared to the PSO+PsA group; ****Significant when compared to the PSO and PsA groups. Agreement was defined by a rating of 6 or 7 on a 7-point Likert scale when asked 'How well do each of the following statements apply to you?'; "neutral" indicates a rating of 3–5 and "disagree" indicates a rating of 1 or 2. Data are reported as percentages of women (the number of respondents selecting each answer divided by the total number of respondents to that question); women were not required to answer every question so missing respondents were not included in proportions. Overall, 573 women were asked this question. PsA: psoriatic arthritis; PSO: psoriasis.

Figure S2. Agreement with statements on the social effects of diagnosis, stratified by time since diagnosis (pooled diagnoses)

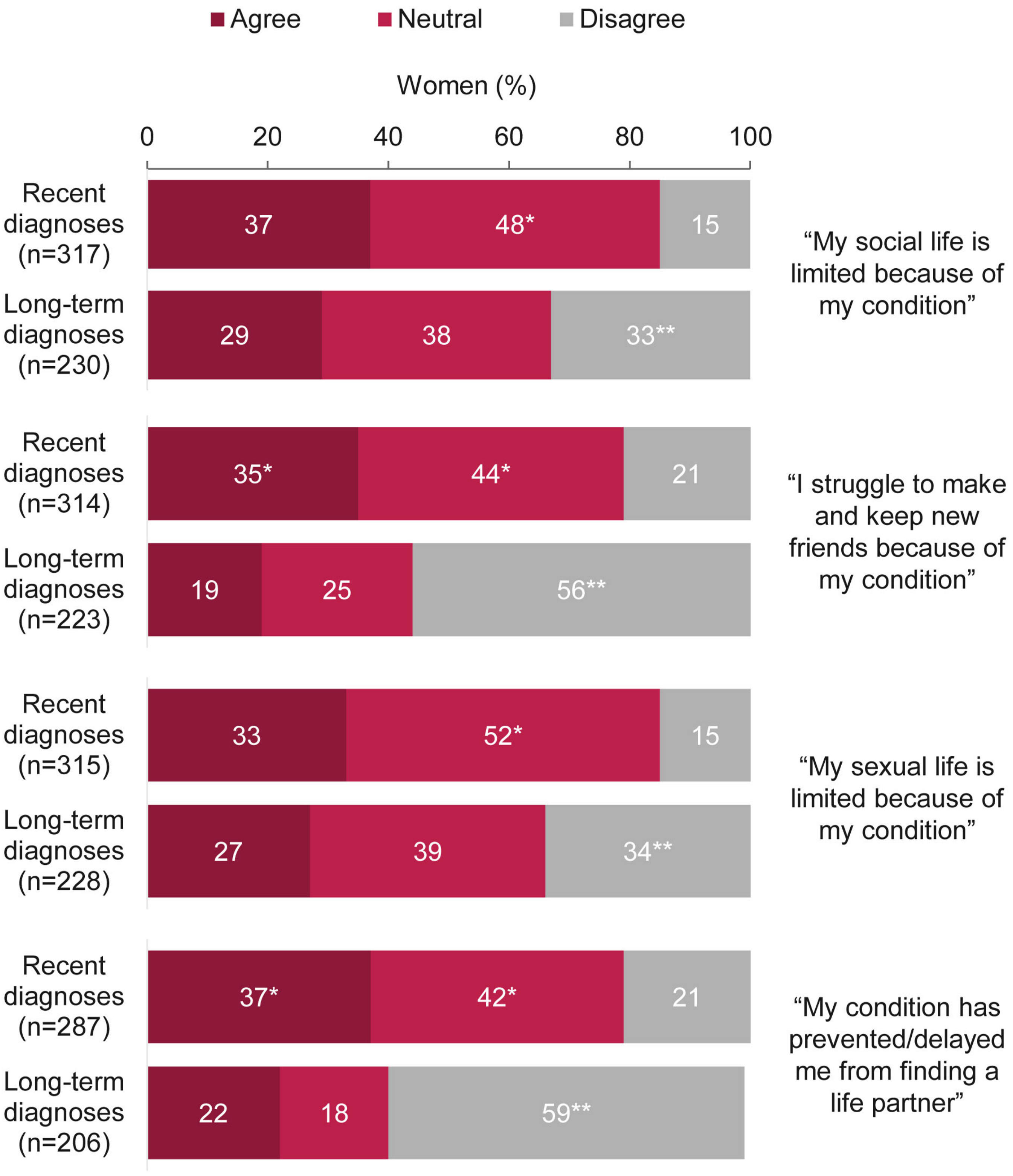

*Significant when compared with the 'long-term diagnoses' group; **Significant when compared with the 'recent diagnoses' group ($p<0.05$). Agreement was defined by a rating of 6 or 7 on a 7-point Likert scale when asked 'How well do each of the following statements apply to you?'; "neutral" indicates a rating of 3–5 and "disagree" indicates a rating of 1 or 2. Recent diagnosis included those diagnosed ≤5 years ago and long-term diagnosis included those diagnosed >5 years ago. Percentages may not add up to 100 due to rounding. Data are reported as percentages of women (the number of respondents selecting each answer divided by the total number of respondents to that question); women were not required to answer every question so missing respondents were not included in proportions. Overall, 573 women were asked this question. PsA: psoriatic arthritis; PSO: psoriasis.

Figure S3. Mean overall mental and physical health scores reported by women at the time of survey, stratified by disease group

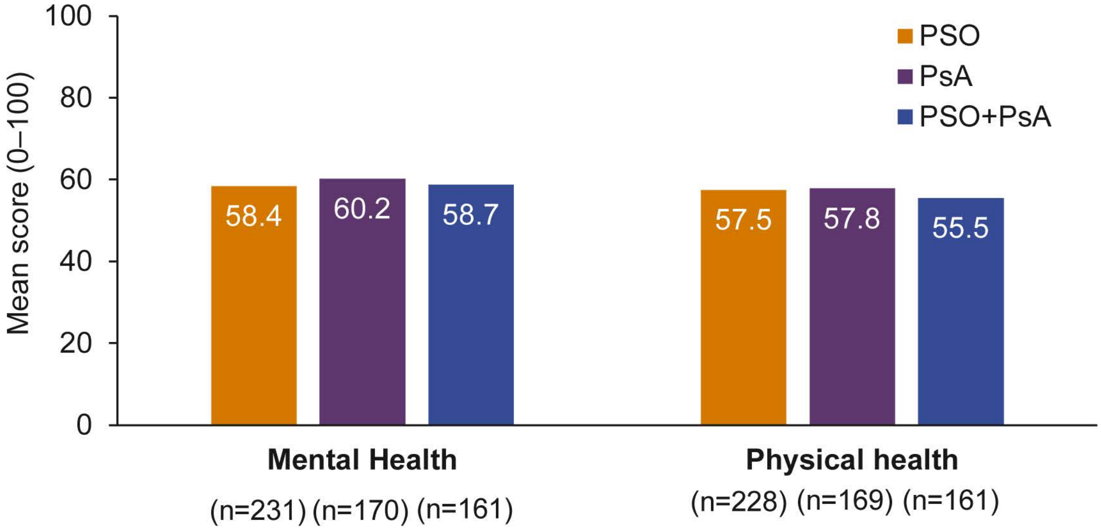

Mental and physical health was rated on a 1–100 scale (whole numbers only), where 100 indicated best health imaginable. Data are reported as percentages of women (the number of respondents selecting each answer divided by the total number of respondents to that question); women were not required to answer every question so missing respondents were not included in proportions. Overall, 573 women were asked these questions. PsA: psoriatic arthritis; PSO: psoriasis.

Figure S4. A) The percentage of women reporting pessimistic and optimistic feelings about the future,[a] and **B)** the percentage of women reporting negative and positive emotions on a normal day and during a disease-flare

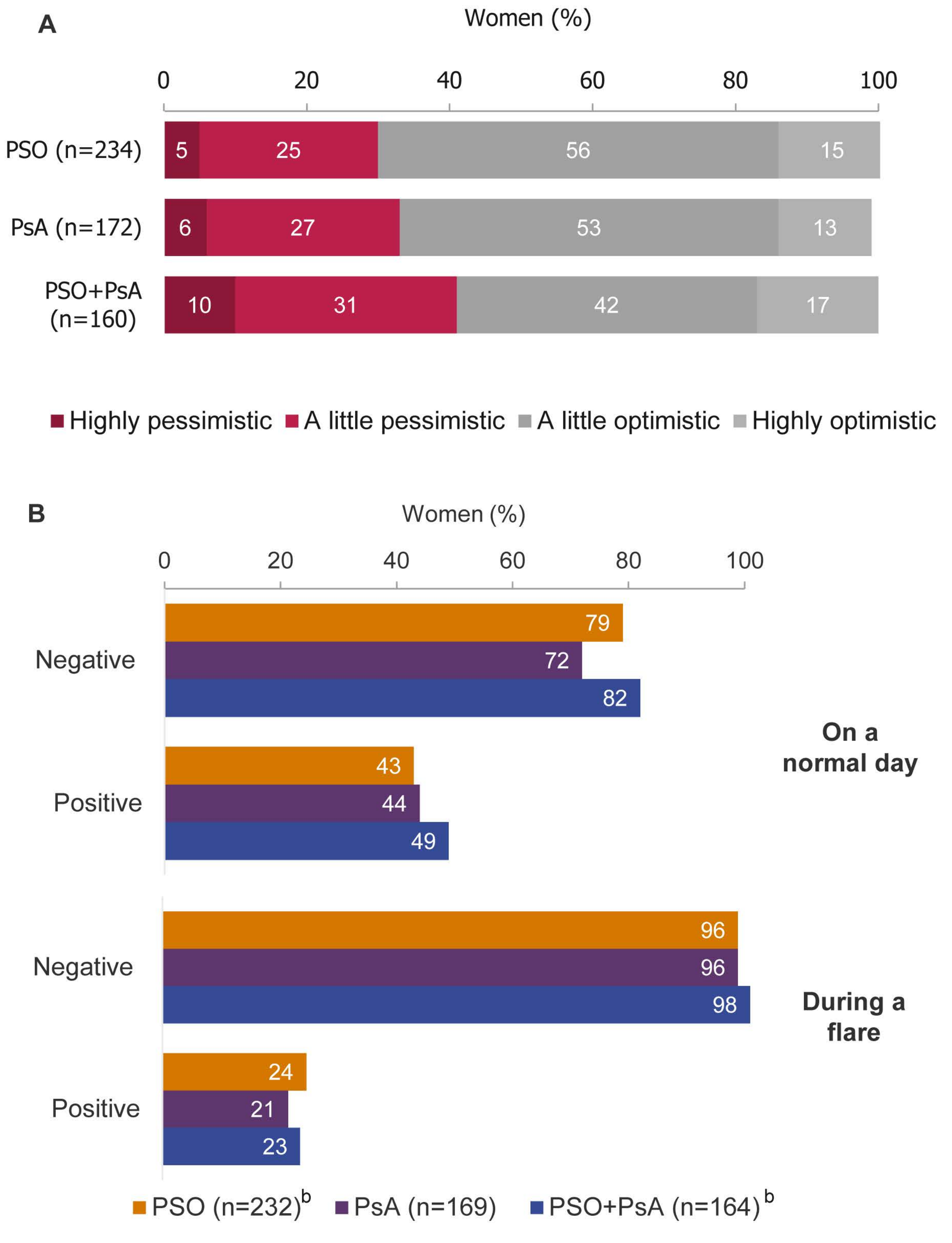

[a]Percentages may not add up to 100 due to rounding. [b]For the PSO and PSO+PsA groups, respondent numbers differed for the 'During a flare' question (PSO [n=233], PSO+PsA [n=163]). Data are reported as percentages of women (the number of respondents selecting each answer divided by the total number of respondents to that question); women were not required to answer every question so missing respondents were not included in proportions. Overall, 573 women were asked these questions. PsA: psoriatic arthritis; PSO: psoriasis.

Figure S5. Employment status by diagnosis (n=573)

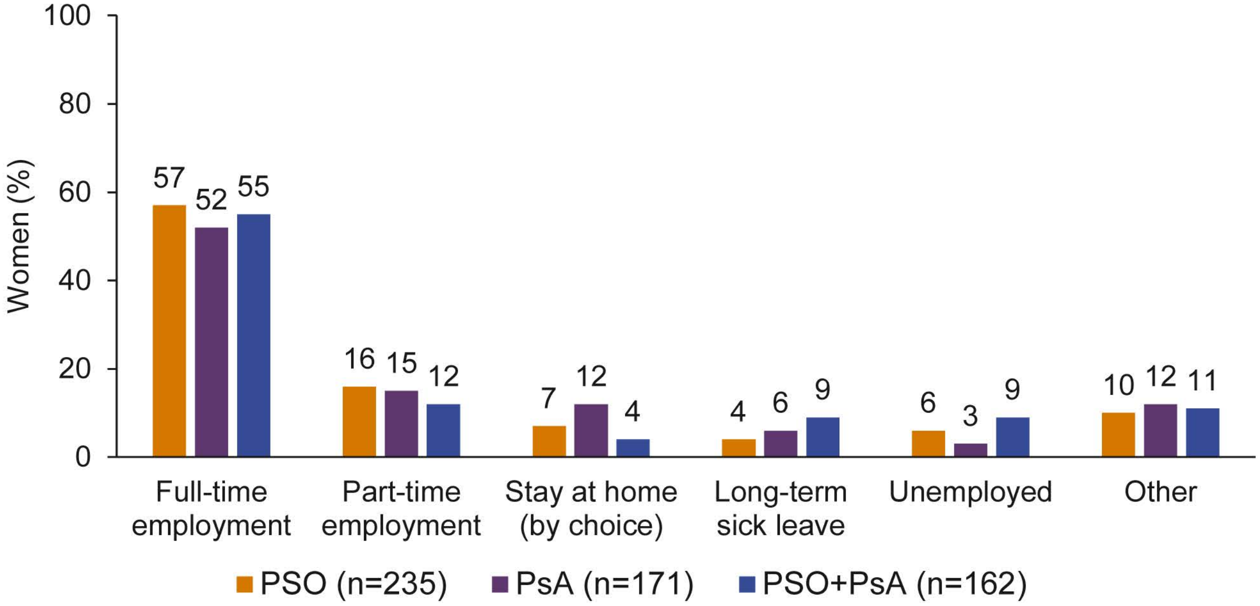

"Other" includes women who reported their employment status as student, self-employed and other. Data are reported as percentages of women (the number of respondents selecting each answer divided by the total number of respondents to that question); women were not required to answer every question so missing respondents were not included in proportions. Women were not required to answer this question. PsA: psoriatic arthritis; PSO: psoriasis.

Figure S6. **A)** The level of support reported by women from the medical professionals managing their condition, both generally and during pregnancy[a] and **B)** The percentage of women requesting access to different support services to help with their condition

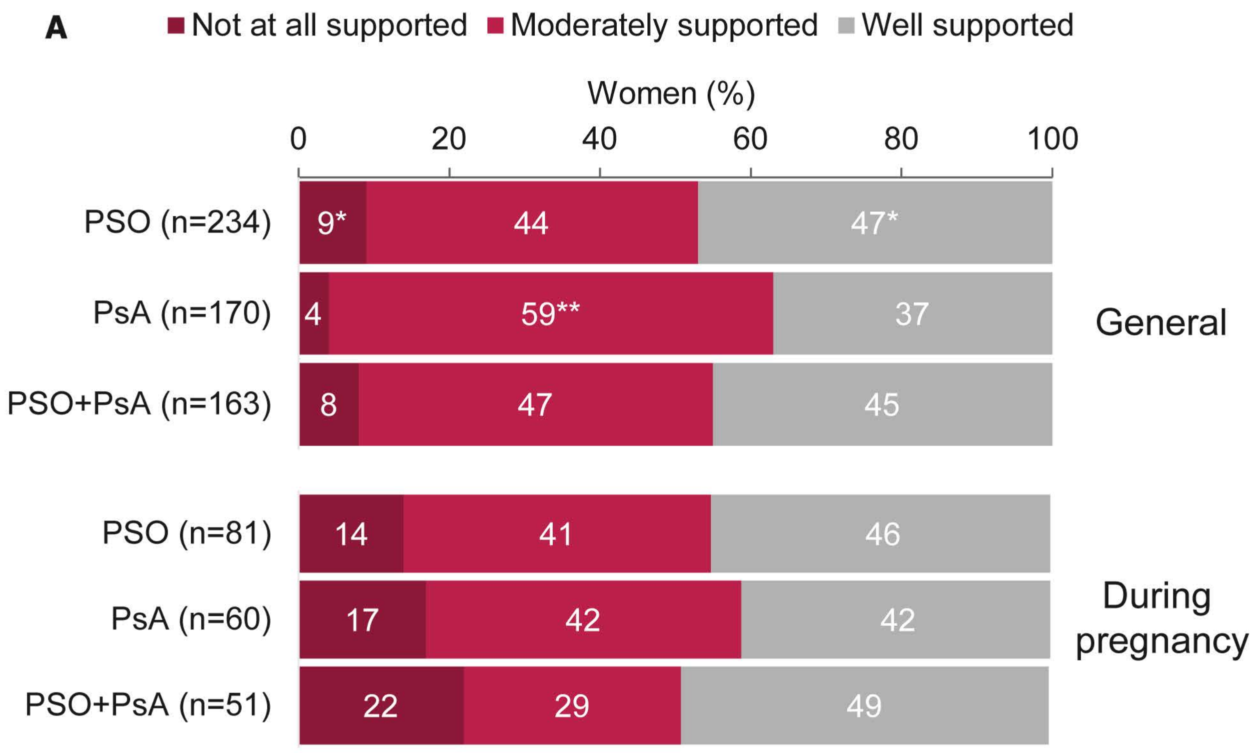

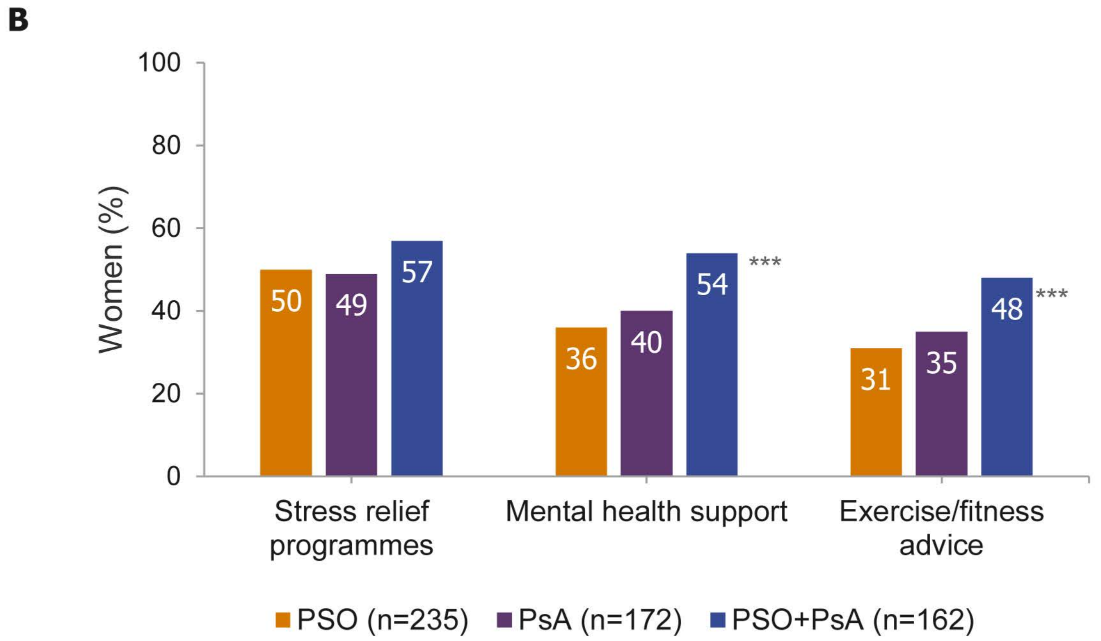

*Significant when compared to the PsA disease group; **Significant when compared to the PSO and PSO+PsA disease group; ***Significant when compared to the PSO and PsA disease groups ($p<0.05$).[a]Women answering the pregnancy-related part of this question were currently pregnant or had given birth in the last five years (n=199); "well supported" was defined by a rating of 6 or 7 on a 7-point Likert scale when asked 'To what extent do you feel you have been supported by doctors and medical professionals?'; "moderately supported" indicates a rating of 3–5 and "not at all supported" indicates a rating of 1 or 2. Percentages may not add up to 100 due to rounding. Data are reported as percentages of women (the number of respondents selecting each answer divided by the total number of respondents to that question); women were not required to answer every question so missing respondents were not included in proportions. PsA: psoriatic arthritis; PSO: psoriasis.

Table S1. Questions and response options from the patient survey

**ELIGIBILITY SECTION**					
S1. Do you or does anyone in your immediate family work in any of the following areas?					
Please select all that apply.					
1. Advertising agency or public relations firm	2. Marketing research firm or market research department of a company	3. Marketing firm or marketing department of a company	4. Newspaper or magazine publishing company	5. Manufacturer, distributor or sales of medications or medical equipment	
6. Physician's office	7. Hospital or healthcare clinic	8.Government health agency	9.None of the above		
S2. Please indicate your gender					
Please select one of the below.					
1. Male	2. Female				
S3. Please indicate your age, and/or the band you fall into					
Please select at least one.					
1. Under 18 years	2. 18–25 years	3. 26–35 years	4. 36–45 years	5. 45+ years	
S4. Which one of these best describes you?					
Please select one.					
1. I have been diagnosed by a doctor as having chronic Plaque Psoriasis	2. I have been diagnosed by a doctor as having chronic Psoriatic Arthritis	3. I have been diagnosed by a doctor as having both Psoriatic Arthritis and Plaque Psoriasis	4. None of the above apply to me		
If both PsA and PsO (S4=3), ask S5a and S5b					
S5a. You said you have been diagnosed with both chronic Plaque Psoriasis and Psoriatic Arthritis. Which were you diagnosed with first?					
Please select one.					
1. Plaque Psoriasis	2. Psoriatic Arthritis	3. Don't know			
S5b. And which do you consider to be your main diagnosis?					
Please select one.					
1. Plaque Psoriasis	2. Psoriatic Arthritis				
S6. When were you first diagnosed with it?					
Please select one.					
1. Less than twelve months ago	2. Twelve months to 3 years ago	3. More than 3 years ago to 5 years ago	4. More than 5 years ago to 10 years ago	5. More than 10 years ago	6. I do not know
S7. To what extent does your chronic Psoriasis or Psoriatic Arthritis impact your daily life?					
Please select one.					
1. Not at all	2. A little	3. A moderate amount	4. A significant amount	5. It impacts everything	
S8. How would your doctor describe the severity on your symptoms?					
Please select one.					
1. Mild	2. Moderate	3. Severe	4. Don't know/Unsure		
S9. And how would you describe the severity of your symptoms?					
Please select one.					
1. Mild	2. Moderate	3. Severe	4. Don't know/Unsure		
S10. Do you experience joint pains?					
Please select one.					

1. Yes, but only mildly or infrequently	2. Yes, frequently or severely	3. No

S11. Please indicate the number of different biologics (injections and infusions) you have been prescribed specifically for your condition				
Please select one.				
1. One	2.Two	3. Three	4. Four	5. Five or more

**Section A: Profiling**					
A1. When were you first prescribed your current biologic medication?					
Please select one.					
1. In the last three months	2. In the last six months	3. In the last year	4. In the last two years	5. Longer ago than this	6. Prefer not to answer

A2. What type of medical professional currently manages your plaque psoriasis/psoriatic arthritis treatment?		
Please select one.		
1. Dermatologist	2. Rheumatologist	3. General Practitioner/Family doctor
4. Nurse	5. Other	6. Prefer not to answer

A3. And what type of doctor first diagnosed you with chronic Plaque Psoriasis/Psoriatic Arthritis?				
Please select one.				
1. Dermatologist	2. Rheumatologist	3. General Practitioner/ Family doctor	4. Other	5. Prefer not to answer

Ask A4 if PSO patient (S4=1 OR S4=3)

A4. Please indicate which parts of your body are currently affected by Plaque Psoriasis in the column on the left. Then for each you select, tell us the severity of it in each of these locations.

**Areas currently affected**	*Please select all that apply*	**Mild**	**Moderate**	**Severe**
Fingernails	□	o	o	o
Palms	□	o	o	o
Other part of hands	□	o	o	o
Elbows	□	o	o	o
Arms	□	o	o	o
Back	□	o	o	o
Buttocks	□	o	o	o
Groin and/or genitals	□	o	o	o
Chest	□	o	o	o
Abdomen	□	o	o	o
Scalp	□	o	o	o
Face	□	o	o	o
Neck	□	o	o	o
Legs	□	o	o	o
Knees	□	o	o	o
Soles of feet	□	o	o	o
Other parts of feet	□	o	o	o
Toenails	□	o	o	o

Prefer not to answer	□

Ask A5 if PsA patient (S4=2 OR S4=3)

A5. And now please indicate which parts of your body you experience Psoriatic Arthritis joint pain in the column on the left. Then for each you select, tell us the severity of it in each of these locations.

**Areas currently affected**	*Please select all that apply*	**Mild**	**Moderate**	**Severe**
Fingers or Hands	□	o	o	o
Neck	□	o	o	o
Arms	□	o	o	o
Elbows	□	o	o	o
Back	□	o	o	o
Hips	□	o	o	o
Groin	□	o	o	o
Knees	□	o	o	o
Heels	□	o	o	o
Feet or Toes	□	o	o	o
Prefer not to answer	□			

A5a. To what extent are each of the following a concern for you?

Please select one option per row.

	**1- Not a concern**	**2**	**3**	**4**	**5 – Great concern**
Itching	o	o	o	o	o
Stinging or Burning	o	o	o	o	o
Pain	o	o	o	o	o
Soreness	o	o	o	o	o
Appearance	o	o	o	o	o
Joint pain	o	o	o	o	o
Cracking or bleeding skin	o	o	o	o	o
Prefer not to answer	□				

A6. How is your Plaque Psoriasis/Psoriatic Arthritis now compared to a year ago?

Please select one.

Much worse now (more sites and more severe)	Somewhat worse now (more sites or more severe)	About the same now	Somewhat better now (fewer sites or less severe)	Much better (fewer sites and less severe)	Prefer not to answer

A7. Please select any other conditions/illnesses that you are/have been diagnosed with.

Please select all that apply.

Diabetes	□	Asthma	□
Depression/anxiety/other mental health issues	□	Osteoporosis	□
High blood pressure/hypertension	□	Inflammatory bowel disease (IBD)	□
Heart disease (including heart failure and experience of heart attack or stroke)	□	Rheumatoid Arthritis	□

Liver disease	◻	Crohn's disease	◻
Kidney disease	◻	Ulcerative colitis	◻
Respiratory conditions (including COPD and Asthma)	◻	Axial spondylarthritis / ankylosing spondylitis	◻
HIV	◻	Osteoarthritis	◻
Sjogren's Disease	◻	Obesity	◻
Dactylitis (inflammation of the fingers or toes)	◻	Cancer	◻
Dyslipidaemia (High cholesterol)	◻	Stomach ulcer	◻
Uveitis/inflammation in the eyes	◻	Multiple Sclerosis	◻
Enthesitis (inflammation of the tendons or ligaments)	◻	Migraine/headaches	◻
SLE (Lupus)	◻	Other (please specify_______)	◻
None of these	◻	Prefer not to answer	◻

A8. How many flares or flare-ups have you experienced in the last 12 months?

A flare up is any worsening of your psoriasis' skin symptoms being either 1) new psoriasis patches in previously uninvolved skin or 2) a worsening of your psoriasis symptoms in skin already affected by psoriasis.

Skin flares/flare-ups:	Prefer not to answer	◻
Joint pain flares/flare-ups:		

Show A9-A12 if A8>0

A9. Thinking about your most recent flare, how in control did you feel?

Please answer using the 7-point scale below.

1 = Not at all in control<br>7 = Fully in control<br>X = Prefer not to answer	Answer:

A10. When was your last flare?

Please select one.

1. Within the last month	2. 1–2 months ago	3. 3–4 months ago	4. 5–6 months ago	5. More than 6 months ago	6. Prefer not to answer

A11. Thinking about this most recent flare, would you say it was:

Please select one.

1. A lot more severe than other flares I've had	2. Slightly more severe than other flares I've had	3. About the same as other flares I've had	4. Slightly less severe than other flares I've had	5. A lot less severe than other flares I've had	6. Prefer not to answer

A12. And in your own words, please tell us what you think triggered your flare on that occasion

Please use the space below for your answer.

◻ Prefer not to answer

1

Section B: Psychological impact of PsO/PsA

B1. Now we would like to know how good or bad your physical health is today. The scale below goes from 0 to 100. 0 means worst health you can imagine and 100 means best health you can imagine.

Please slide the scale to indicate how your health is today.

Worst physical health I can imagine	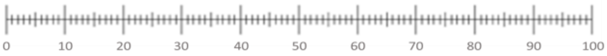	Best physical health I can imagine

□ Prefer not to answer

B2. And now, thinking about your mental health today, where on the scale would you put yourself?

Please slide the scale to indicate how your mental health is today.

Worst mental health I can imagine	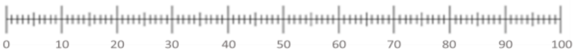	Best mental health I can imagine

□ Prefer not to answer

B3. How optimistic do you feel about the future?

Please select one.

1.Highly optimistic	2. A little optimistic	3. A little pessimistic	4. Highly pessimistic	5. Prefer not to answer

B4. How does living with Plaque Psoriasis/Psoriatic Arthritis make you feel?

Please select any of the below that apply to you.

	**On a normal day**	**When I flare/flare-up**
Irritated	o	o
Frustrated	o	o
Angry	o	o
Ashamed	o	o
Unattractive	o	o
Worried	o	o
Tired	o	o
Sad	o	o
Depressed	o	o
Unwell	o	o
Lonely	o	o
Anxious	o	o
Isolated	o	o
Resigned	o	o
Overwhelmed	o	o
Resilient	o	o
Courageous	o	o
Other (specify)		
None of these	o	o

□ Prefer not to answer

B5. Please take a look at these two contrasting statements. Please indicate where you personally fit between the two.

Please use the scale to select one.				
My Plaque Psoriasis/Psoriatic Arthritis affects only my skin	2	3	4	My Plaque Psoriasis/Psoriatic Arthritis affects my whole body (inside my body and outside on my skin
□	□	□	□	□
□ Prefer not to answer				
B6. Here are another two contrasting statements. Please indicate where you personally fit between the two.				
Please use the scale to select one.				
I am full in control of my Plaque Psoriasis/Psoriatic Arthritis	2	3	4	My Plaque Psoriasis/Psoriatic Arthritis is fully in control of me
□	□	□	□	□
□ Prefer not to answer				

1

**Section C: Impact of PsO/PsA on daily life**					
C1: Below is a list of common things that people might go through. You may have experienced some or all of these already, others might not (yet) be relevant.					
For each you have experienced, please select how stressed you felt at each of these.					
	**Not particularly stressful**	**A little stressed**	**Moderately stressed**	**Severely stressed**	**Does not apply to me**
Being at school/in education	o	o	o	o	o
Studying for exams	o	o	o	o	o
Losing a loved one	o	o	o	o	o
Attending job interviews/starting a new job	o	o	o	o	o
Moving house	o	o	o	o	o
Marriage	o	o	o	o	o
Divorce or ending a long term relationship	o	o	o	o	o
Being out of work	o	o	o	o	o
Financial hardship	o	o	o	o	o
Dating/finding a partner	o	o	o	o	o
Trying to conceive	o	o	o	o	o
Becoming a new parent	o	o	o	o	o
Breastfeeding	o	o	o	o	o
□ Prefer not to answer					

C2. And now tell us what kind of impact your Plaque Psoriasis/Psoriatic Arthritis had at each of these points in time.					
For each you have experienced, please select how much of an impact you felt at each of these.					
	**My condition had no impact**	**More frequent flares**	**More severe flares**	**Worsening of symptoms**	**Did not have Psoriasis/PsA at this time**
Being in school/in education	o	o	o	o	o
Studying for exams	o	o	o	o	o
Losing a loved one	o	o	o	o	o
Attending job interviews/starting a new job	o	o	o	o	o
Moving house	o	o	o	o	o
Marriage	o	o	o	o	o
Divorce or ending a long term relationship	o	o	o	o	o
Being out of work	o	o	o	o	o
Financial hardship	o	o	o	o	o
Dating/finding a partner	o	o	o	o	o
Trying to conceive	o	o	o	o	o
Becoming a new parent	o	o	o	o	o
Breastfeeding	o	o	o	o	o
◻ Prefer not to answer					

C3. How well do each of the following statements apply to you?							
Please answer using a scale 1 to 7, where 1 is "Does not describe me at all" and 7 is "Describes me very well".							
I have to limit the types and colours of clothing I wear	1	2	3	4	5	6	7
My social life is limited because of my PSO/PsA	1	2	3	4	5	6	7
I struggle to make and keep new friends because of my PSO/PsA	1	2	3	4	5	6	7
My sexual life is limited because of my PSO/PsA	1	2	3	4	5	6	7
My condition has created problems or arguments with my partner or close family members	1	2	3	4	5	6	7
My loved ones don't understand enough about PSO/PsA	1	2	3	4	5	6	7

My condition has prevented me/delayed me finding a life partner	1	2	3	4	5	6	7
My condition had an impact on the number of children I had, or am planning to have	1	2	3	4	5	6	7
My condition stops me from being the mum I want to be	1	2	3	4	5	6	7
I try to hide my condition from other people	1	2	3	4	5	6	7
I have had relationships end because my friends, spouse or significant other does not accept my condition	1	2	3	4	5	6	7
I feel stigmatised because of my condition	1	2	3	4	5	6	7
I don't understand enough about my condition	1	2	3	4	5	6	7
It seems that, because of my condition, I'm not 'me' anymore	1	2	3	4	5	6	7
I am worried that my children may inherit my condition	1	2	3	4	5	6	7
I worry about my ability to cope with the administrative tasks and paperwork related to managing my disease (e.g. insurance forms, retirement accounts)	1	2	3	4	5	6	7
I am afraid of going near other people who might be ill, due to my weak immune system	1	2	3	4	5	6	7
◻ Prefer not to answer							
C4. On what occasions, if any, do you feel particularly stigmatised or unfairly marked out by your Plaque Psoriasis/Psoriatic Arthritis?							

Please answer in the space below.
☐ Prefer not to answer

1

**Section D: Impact of PsO/PsA treatments on daily life**							
D1**.** How well do each of the following statements about your treatment apply to you?							
Please answer using a scale of 1 to 7, where 1 is "Does not describe me at all", and 7 is "Describes me very well".							
I feel embarrassed by the process of my treatment	1	2	3	4	5	6	7
My treatment enables me to live a normal life	1	2	3	4	5	6	7
Beyond my medication, I'm not sure what else I can do, or what other possibilities are open to me on top of medication	1	2	3	4	5	6	7
I am well aware of the support services and help options available to me	1	2	3	4	5	6	7
I worry about the long-term impact of taking my treatment	1	2	3	4	5	6	7
My treatment regularly causes me pain or discomfort	1	2	3	4	5	6	7
☐ Prefer not to answer							
D2. And how well do each of the following statements about your relationship with the medical team that treats you?							
Please answer using a scale of 1 to 7, where 1 is "Does not describe me at all" and 7 is "Describes me very well".							
I have confidence in the doctor(s) treating and managing my condition	1	2	3	4	5	6	7
My doctor(s) and their team helped me understand the emotional impact of living with my condition	1	2	3	4	5	6	7
My doctor(s) and their team are honest with me about what I can realistically expect from my treatment	1	2	3	4	5	6	7
I have enough face-to-face time with the doctor(s) treating my condition	1	2	3	4	5	6	7

When I started my current treatment, I was given all the information and support that I needed	1	2	3	4	5	6	7
When my doctor was explaining my treatment to me, I had the opportunity to ask questions about my condition and treatment	1	2	3	4	5	6	7
If my treatment started affecting me in an undesirable way, I'd immediately tell my doctor	1	2	3	4	5	6	7
My doctors and their teams are working well together to share relevant information about my health and treatment with me	1	2	3	4	5	6	7
My doctor and I agree on what we are aiming to achieve	1	2	3	4	5	6	7
My doctor and I are both equally involved in the decisions made about my treatment	1	2	3	4	5	6	7
◻ Prefer not to answer							

D3. Roughly speaking, how often do you forget to take your treatment/miss a dose?

Please select one.

1. Frequently	2. Sometimes	3. Very occasionally	4. Never or almost never	5. Prefer not to answer

D4. Have you ever decided to stop or pause your biologic treatment temporarily, or considered doing this?

Please select one.

1. Yes – I stopped my biologic treatment for a time	2. No, but I seriously considered it	3. No, and have not considered doing so	4. Prefer not to answer

Show D5 if D4=1

D5. Did you tell your doctor you had stopped your treatment?

Please select one.

1. Yes	2. No	3. Prefer not to answer

If stopped treatment (D4=1), ask D6

D6. What circumstances led you to stopping your treatment temporarily?

Please select all that apply.

My side-effects became hard to tolerate	◻
My condition worsened	◻
My condition improved (for example there are no symptoms visible on my skin)	◻
I was trying to conceive/trying for a baby	◻

I was pregnant	□
I was breastfeeding	□
I needed to save money or could not afford treatment	□
I had surgery	□
I wanted to go on holiday/needed to travel abroad	□
Other (please specify)	
□ Prefer not to answer	
If not yet stopped treatment (D4=2 or 3), ask D7	
D7. In what circumstances, if any, would you consider stopping your treatment temporarily	
Please select all that apply.	
If the side-effects became hard to tolerate	□
If my condition worsened	□
If my conditioned improved (for example there are no symptoms visible on my skin)	□
To try to conceive/to try for a baby	□
If I was pregnant	□
To breastfeed	□
If I needed to save money	□
If I planned to have surgery	□
If I wanted to go on holiday/needed to travel abroad	□
Other (please specify)	
□ Prefer not to answer	

D8. To what extent, do you feel you have been well supported by doctors and medical professionals?							
Please answer using a scale of 1 to 7, where 1 is "Not well supported at all", and 7 is "Supported very well".							
1	2	3	4	5	6	7	Prefer not to answer
If scoring 1–4 at D8, ask D9							
D9. Why do you say that?							
Please answer in the space below.							
□ Prefer not to answer							

1

**Section E: Impact on professional life and finances**	
E1. Which of the following best describe your current employment status?	
Please select one.	
Employed full time on an ongoing basis	□
Employed part time or on a short-term contract	□
Housewife/choose to stay at home	□
Unemployed and looking for employment	□
Student	□
On long-term sick leave or disability	□
Self-employed	□
Other	□
□ Prefer not to answer	
E2. In total, how long have you spent to date in employment?	

Please answer in the space below.							
________ years							
□ Prefer not to answer							
Show E3 only if codes 1, 2, 7 at E1							
E3. Briefly tell us what you do for a living							
Please answer in the space below.							
□ Prefer not to answer							
E4. What proportion of your working life so far has been spent:							
Please fill in the percentages below.							
In employment	%						
Out of work through my own choosing (e.g. maternity leave, career breaks)	%						
Out of work not through my own choosing (e.g. unemployment, enforced absence, sick leave)	%						
□ Prefer not to answer							
E5. Which of these best describes you?							
Please select one.							
I have met or exceeded my professional ambitions	□						
I have not had the career I wanted – but this is not because of my condition	□						
I have not had the career I wanted – and my condition is a major factor in preventing me having that career	□						
I didn't have any professional ambitions	□						
□ Prefer not to answer							
E6. In a typical year, how many days do you take off sick, as a result of your Plaque Psoriasis/Psoriatic Arthritis?							
Please answer in the space below.							
______________days per year							
□ Prefer not to answer							
E7. Has your condition limited your choice of occupation? If so, in what ways?							
Please answer in the space below.							
□ Prefer not to answer							
E8. How well do each of the following statements describe you?							
Please answer using a scale of 1 to 7, where 1 is "Does not describe me at all" and 7 is "Describes me very well".							
I am happy overall with my career	1	2	3	4	5	6	7
If I didn't suffer with [Plaque Psoriasis/Psoriatic Arthritis], I would have better career prospects	1	2	3	4	5	6	7

I feel discriminated against at work because of my condition	1	2	3	4	5	6	7
I worry that my job is at risk because of my condition	1	2	3	4	5	6	7
I earn less than colleagues doing the same job as me because of my condition	1	2	3	4	5	6	7
I am less productive than my colleagues because of my condition	1	2	3	4	5	6	7
I have had to take on debt or loans to make ends meet, because my condition has stopped me earning enough	1	2	3	4	5	6	7
The uniform or work clothing I have to wear in the workplace makes my symptoms worse	1	2	3	4	5	6	7
The environment or working conditions have an adverse effect on my condition	1	2	3	4	5	6	7

In the workplace, I have to ask other people to do some things for me because my condition restricts what I can do	1	2	3	4	5	6	7
Career paths and promotions are limited for me because of my condition	1	2	3	4	5	6	7
◻ Prefer not to answer							

E9. Here are two contrasting statements. Please indicate where you personally fit between the two				
Please use the scale to indicate where you fit between the two.				
1. I am fully in control of my work life	2	3	4	5. My Plaque Psoriasis/Psoriatic Arthritis dictates my work life
◻ Prefer not to answer				

E10. Have you ever…		
Please choose yes or no for each.		
Been turned down for a job directly because of your condition	o Yes	o No
Lost a job directly because of your condition	o Yes	o No
Been denied a promotion directly because of your condition	o Yes	o No
None of the above apply to me		
◻ Prefer not to answer		

1

**SECTION F: Family Planning**					
F1. Which one of the following statements best describes you currently?					
Please select one.					
1. I am trying to conceive or expect to start trying soon	2. I am currently pregnant	3. I have given birth in the last year	4. I have given birth in the last 2-5 years	5. None of the above	6. Prefer not to answer
If pregnant or given birth (2–4 at F1), ask F2					
F2. Was your pregnancy:					
Please select one.					
1. Planned	2. Unintended	3. Prefer not to answer			
F3. How many children do you have, in each of the following age bands?					
Please fill in the below.					
Age 0 to 5:	Age 6 to 10:	Age 11 to 17:			

No children:	Prefer not to answer:		
F4. Which one of these statements best describes you?			
Please select one.			
1. I am planning to get pregnant in the next year	2. I am planning to get pregnant in the next 2–3 years	3. I have no plans to get pregnant, but expect it to happen at some point	4. I have decided not to have any (more) children
□ Prefer not to answer			

F5. How well do each of the following statements describe you?								
Please answer using a scale of 1 to 7, where 1 is "Does not describe me at all." and 7 is "Describes me very well".								
I am worried about the impact of my medication on my fertility	1	2	3	4	5	6	7	Does not apply
I am worried that my medication could have an effect on my unborn child	1	2	3	4	5	6	7	Does not apply
I'm worried that my condition might mean being unable to look after my new baby as well as I'd like	1	2	3	4	5	6	7	Does not apply
I have decided to have a smaller family – or even no children at all – because of my condition	1	2	3	4	5	6	7	Does not apply
I am more likely to pursue adoption because of my condition	1	2	3	4	5	6	7	Does not apply
My partner is worried about the impact of my condition on the pregnancy	1	2	3	4	5	6	7	Does not apply
I feel that I won't be able to breast feed because of my condition	1	2	3	4	5	6	7	Does not apply
I have had arguments and conflict with my partner specifically about my condition and treatment, and becoming pregnant	1	2	3	4	5	6	7	Does not apply
I feel I can't experience the same kind of pregnancy that women who don't have my condition would have	1	2	3	4	5	6	7	Does not apply

□ Prefer not to answer

Ask F6 if trying to conceive (F1=1)

F6. In what ways, if any, have you changed your approach to managing your condition because you are trying for a baby?

Please select any that apply to you.

I have stopped my treatment	□
I have reduced the dose or frequency of my treatment	□
I have switched to another treatment on the recommendation of a doctor	□
I have made diet or lifestyle changes	□
I have done something else (please specify)	
No, I have not changed by approach	□

□ Prefer not to answer

Ask F7 if trying to conceive or planned pregnancy (F1=1 or F2=1)

F7. Did you consult a doctor or other medical professional once you had decided to try for a baby (and prior to becoming pregnant)?

Please select one.

1. Yes	2. No	3. Prefer not to answer

Ask F8 if trying to conceive or planned pregnancy (F1=1 or F2=1)

F8. What information sources did you use to find out more about managing your condition while trying for a baby?

Please answer in the space below.

□ Prefer not to answer

Ask F9 if trying to conceive or planned pregnancy (F1=1 or F2=1)

F9. Did your partner or other family member or close friend find and share information with you?

Please select one.

1. Yes	2. No	3. Prefer not to answer

Show F10-F11 only if code 1 selected at F9

Ask F10 if trying to conceive or planned pregnancy (F1=1 or F2=1)

F10. What did they share with you?

Please answer in the space below.

□ Prefer not to answer

Ask F11 if trying to conceive or planned pregnancy (F1=1 or F2=1)

F11. And how useful was that information?

1. Very useful	2. Fairly useful	3. Not useful	4. Prefer not to answer

1

Section G: Pregnancy Journey

ASK SECTION G IF CURRENTLY PREGNANT OR HAVE GIVEN BIRTH IN LAST 5 YEARS (F1=2, 3 OR 4), OTHERWISE SKIP TO SECTION H

G1. Please think back to when you discovered you were (last) pregnant. Which of these words describe how you felt?			
Please select all that apply.			
1. Irritated	o	2. Frustrated	o
3. Angry	o	4. Ashamed	o
5. Unattractive	o	6. Worried	o
7. Tired	o	8. Sad	o
9. Depressed	o	10. Unwell	o
11. Lonely	o	12. Anxious	o
13. Isolated	o	14. Resigned	o
15. Pleased	o	16. Joyous	o
17. Excited	o	18. Relieved	o
19. Shocked	o	20. Overwhelmed	o
21. Scared	o	22. Other (specify:____)	o
23. None of these	o	24. Prefer not to answer	o

G2. Thinking of your pregnancy 'journey', how well supported did you feel at each of these stages?			
Please select one answer for each stage.			
**While thinking of starting a family**			
Very well supported	Somewhat well supported	Not very well supported	Not supported at all
**While trying to conceive**			
Very well supported	Somewhat well supported	Not very well supported	Not supported at all
**In the first trimester**			
Very well supported	Somewhat well supported	Not very well supported	Not supported at all
**In the second trimester**			
Very well supported	Somewhat well supported	Not very well supported	Not supported at all
**In the third trimester**			
Very well supported	Somewhat well supported	Not very well supported	Not supported at all
**During birth**			
Very well supported	Somewhat well supported	Not very well supported	Not supported at all
**In the immediate days following the birth** (show only to those having given birth: F1=3 or 4)			
Very well supported	Somewhat well supported	Not very well supported	Not supported at all
**In the weeks and months following birth** (show only to those having given birth: F1=3 or 4)			
Very well supported	Somewhat well supported	Not very well supported	Not supported at all
□ Prefer not to answer			

G3. And how satisfied were you that the different medical professionals you saw while pregnant communicated with each other and understood your case?				
Please select one.				
1. Very satisfied	2. Fairly satisfied	3. Fairly dissatisfied	4. Very dissatisfied	5. Prefer not to answer
If dissatisfied (G3=3 or 4), ask G4				

G4. Why were you not satisfied?
Please answer in the space below.
□ Prefer not to answer

G5. Thinking about your pregnancy, how well supported did you feel, by each of the following?							
Please use the 7-point scale below, where 1 means you had little or no support and 7 means you had all the support you needed.							
The doctor or medical professional(s) managing your psoriasis/psoriatic arthritis	1	2	3	4	5	6	7
The doctor or medical professional(s) managing your pregnancy	1	2	3	4	5	6	7
Your partner	1	2	3	4	5	6	7
□ Prefer not to answer							

G6. Did you do any of the follow while pregnant?	
Please select all that apply.	
Stop your <u>biologic</u> treatment for your [Plaque Psoriasis/Psoriatic Arthritis] on medical advice	Reduce the dose or frequency of your biologic treatment on medical advice
Decide yourself to stop your <u>biologic</u> treatment for your [Plaque Psoriasis/Psoriatic Arthritis]	Decide yourself to reduce the dose or frequency of your biologic treatment
Switch to another treatment for your [Plaque Psoriasis/Psoriatic Arthritis] on medical advice	Make diet or lifestyle changes on medical advice
Decide yourself to switch to another treatment for your [Plaque Psoriasis/Psoriatic Arthritis]	Decide yourself to make diet or lifestyle changes
None of these	Prefer not to answer

G7. What information sources did you use to find out more about managing your condition while pregnant?
Please answer in the space below.
□ Prefer not to answer

G8. During your pregnancy, how many flares or flare-ups did you have?
A flare-up is any worsening of your psoriasis' skin symptoms being either 1) new psoriasis patches in previously uninvolved skin or 2) a worsening of your psoriasis symptoms in skin already affected by psoriasis
______________________skin flares or flare-ups during pregnancy
______________________joint pain flares or flare-ups during pregnancy
□ Prefer not to answer
Ask G9 if >0 flares at G8

G9. And were those flares:			
Please select one.			
A lot worse than normal	o	A little worse than normal	o
About the same as normal	o	A little less severe than normal	o
A lot less severe than normal	o	Prefer not to answer	o

Ask G10 if >0 flares at G8

G10. And during these flares, did you feel:			
Please select one.			
A lot more worried than normal	o	A little more worried than normal	o
No more worried than normal	o	A little less worried than normal	o
A lot less worried than normal	o	Prefer not to answer	o

Do not show G11 if F3 “no children” or “prefer not to answer”

G11. Did you experience flares after being pregnant?		
1. Yes	2. No	3. Prefer not to answer

Ask G12 if flared after pregnancy (G11=1)

G12. Thinking about your post-pregnancy flares, how severe were these compared to flares before pregnancy? Were they:			
Please select one.			
A lot more severe than pre-pregnancy flares	o	Slightly more severe than pre-pregnancy flares	o
About the same as my pre-pregnancy flares	o	Slightly less severe than pre-pregnancy flares	o
A lot less severe than pre-pregnancy flares	o	Prefer not to answer	o

If currently pregnant (F1=2), ask G13

G13. Are you intending to breastfeed?		
Please select one.		
1. Yes	2. No	3. Prefer not to answer

If given birth in the last year (F1=3), ask G14

G14. Did you breastfeed?		
Please select one.		
1. Yes	2. No	3. Prefer not to answer

G15. How well do each of the following statements describe you, relating to how you felt post-pregnancy?							
Please answer using a scale of 1 to 7, where 1 is “Does not describe me at all” and 7 is “Describes me very well”.							
I was worried about experiencing more flares/ flare-ups or more severe flaring after giving birth	1	2	3	4	5	6	7
I was worried that my condition would prevent me from being the mum I want to be	1	2	3	4	5	6	7

I was given enough information to manage my condition post-pregnancy	1	2	3	4	5	6	7
My partner lacked enough information and support to help me post-pregnancy	1	2	3	4	5	6	7
I experienced post-natal depression, or felt depressed after having given birth	1	2	3	4	5	6	7
◻ Prefer not to answer							

G16. Have your experiences through your pregnancy [and post-pregnancy] made you:			
Please select one.			
More likely to try for another child	o	Less likely to try for another child	o
No more or less likely to try for another child	o	Prefer not to answer	o

1

**Section H: Demographics and wrap-up**			
H1. Which of the following support services would you like to have, or be available to you, to give you more help in managing and living with your condition?			
Please select all that apply.			
Mental health support (such as psychological therapy)	o	Meditation techniques	o
Stress relief programmes, such as sophrology or yoga	o	Exercise and fitness advice	o
Counselling	o	Careers advice	o
Support groups with other Psoriatic disease patients	o	Advice on explaining your disease to other people	o
◻ None of these			
◻ Prefer not to answer			
H2. What [Plaque Psoriasis / Psoriatic Arthritis] associations are you a member of?			
Please answer in the space below.			
◻ Prefer not to answer			

H3. What is your marital status?	
Please select one.	
Single	◻
Married/registered partnership	◻
Divorced	◻
Separated	◻
Widowed	◻
Cohabiting/living with someone	◻
Prefer not to answer	◻
H4. Who is currently living in the household with you?	
Please select all that apply	
Spouse	◻

Child/children	□
I live alone	□
Parent(s)	□
Sibling	□
Other care giver	□
Friend	□
Roommate, housemate or lodger	□
Prefer not to answer	□
H5. Do you currently live:	
Please select one.	
In a village or rural area	□
In a small town	□
In a large town	□
In the suburbs of a large city	□
In the middle of a large city	□
Prefer not to answer	□
H6. What is the highest level of school you have completed?	
Please select one.	
Attended school up to age 16 or younger	□
Attended school up to age 18 or younger	□
Professional or technical training after completing school	□
Attend university but not graduated	□
Bachelor’s degree or college degree	□
Master’s degree or higher	□
Prefer not to answer	□
H7. Approximately, which of the following options best reflects your annual total household income?	
Please select one.	
Up to £10,000	□
£10,000–£19,999	□
£20,000–£29,999	□
£30,000–£39,999	□
£40,000–£49,999	□
£50,000–£59,999	□
£60,000–£69,999	□
£70,000–£79,999	□
£80,000–£89,999	□
£90,000–£99,999	□

£100,000–£109,999	◻
£110,000–£119,999	◻
£120,000–£129,999	◻
£130,000–£139,999	◻
£140,000–£149,999	◻
£150,000 or more	◻
Prefer not to answer	◻

Please note that this is a re-drawn representation of survey questions, so formatting differs to the original online survey.
